# Liraglutide Reduces Vascular Damage, Neuronal Loss, and Cognitive Impairment in a Mixed Murine Model of Alzheimer’s Disease and Type 2 Diabetes

**DOI:** 10.3389/fnagi.2021.741923

**Published:** 2021-12-16

**Authors:** Maria Jose Carranza-Naval, Angel del Marco, Carmen Hierro-Bujalance, Pilar Alves-Martinez, Carmen Infante-Garcia, Maria Vargas-Soria, Marta Herrera, Belen Barba-Cordoba, Isabel Atienza-Navarro, Simon Lubian-Lopez, Monica Garcia-Alloza

**Affiliations:** ^1^Division of Physiology, School of Medicine, Universidad de Cádiz, Cádiz, Spain; ^2^Instituto de Investigacion e Innovacion en Ciencias Biomedicas de la Provincia de Cádiz (INIBICA), Cádiz, Spain; ^3^Salus Infirmorum-Universidad de Cádiz, Cádiz, Spain; ^4^Section of Neonatology, Division of Pediatrics, Hospital Universitario Puerta del Mar, Cádiz, Spain

**Keywords:** Alzheimer’s disease, type 2 diabetes, liraglutide, neuronal loss, hemorrhage, inflammation

## Abstract

Alzheimer’s disease is the most common form of dementia, and epidemiological studies support that type 2 diabetes (T2D) is a major contributor. The relationship between both diseases and the fact that Alzheimer’s disease (AD) does not have a successful treatment support the study on antidiabetic drugs limiting or slowing down brain complications in AD. Among these, liraglutide (LRGT), a glucagon-like peptide-1 agonist, is currently being tested in patients with AD in the Evaluating Liraglutide in Alzheimer’s Disease (ELAD) clinical trial. However, the effects of LRGT on brain pathology when AD and T2D coexist have not been assessed. We have administered LRGT (500 μg/kg/day) to a mixed murine model of AD and T2D (APP/PS1xdb/db mice) for 20 weeks. We have evaluated metabolic parameters as well as the effects of LRGT on learning and memory. *Postmortem* analysis included assessment of brain amyloid-β and tau pathologies, microglia activation, spontaneous bleeding and neuronal loss, as well as insulin and insulin-like growth factor 1 receptors. LRGT treatment reduced glucose levels in diabetic mice (db/db and APP/PS1xdb/db) after 4 weeks of treatment. LRGT also helped to maintain insulin levels after 8 weeks of treatment. While we did not detect any effects on cortical insulin or insulin-like growth factor 1 receptor m-RNA levels, LRGT significantly reduced brain atrophy in the db/db and APP/PS1xdb/db mice. LRGT treatment also rescued neuron density in the APP/PS1xdb/db mice in the proximity (*p* = 0.008) far from amyloid plaques (*p* < 0.001). LRGT reduced amyloid plaque burden in the APP/PS1 animals (*p* < 0.001), as well as Aβ aggregates levels (*p* = 0.046), and tau hyperphosphorylation (*p* = 0.009) in the APP/PS1xdb/db mice. Spontaneous bleeding was also ameliorated in the APP/PS1xdb/db animals (*p* = 0.012), and microglia burden was reduced in the proximity of amyloid plaques in the APP/PS1 and APP/PS1xdb/db mice (*p* < 0.001), while microglia was reduced in areas far from amyloid plaques in the db/db and APP/PS1xdb/db mice (*p* < 0.001). This overall improvement helped to rescue cognitive impairment in AD-T2D mice in the new object discrimination test (*p* < 0.001) and Morris water maze (*p* < 0.001). Altogether, our data support the role of LRGT in reduction of associated brain complications when T2D and AD occur simultaneously, as regularly observed in the clinical arena.

## Introduction

Age remains the main risk factor for Alzheimer’s disease (AD). Nevertheless, metabolic disorders, and type 2 diabetes (T2D) specifically, may also increase the risk of AD by over twofolds (Sims-Robinson et al., 2010; Ryu et al., 2019). Likewise, the close relationship between AD and T2D has been long analyzed (Biessels and Despa, 2018; Vieira et al., 2018). Studies on animal models have shown that metabolic alterations may affect different amyloid species and amyloid deposition, although the effects seem to be highly dependent on the specific animal model used (Ramos-Rodriguez et al., 2014; Wang et al., 2014; Chatterjee and Mudher, 2018; Natunen et al., 2020). On the other hand, while it is widely accepted that cognitive dysfunction is an important and common comorbidity of diabetes, this effect does not seem to be directly related to alterations in amyloid pathology, and previous studies have reported no differences in cerebrospinal fluid amyloid-β (Aβ)-42 levels, global or regional AD pathology, or amyloid burden (Arvanitakis et al., 2006; Moran et al., 2015; Pruzin et al., 2017; Biessels and Despa, 2018). Interestingly, a recent study using PET with ^18^F-Florbetaben for global Aβ standardized value uptake ratio shows that prediabetes, but not T2D, is associated with higher Aβ levels (Luchsinger et al., 2020), supporting further studies on the effects of metabolic disorders and T2D treatments at this level. It has also been suggested that the inconsistent findings relating T2D to AD pathology might be due to the inclusion of heterogeneous diabetic populations and not accounting for glycemic control. In this sense, individuals with undiagnosed diabetes have increased risk of dementia when compared with individuals with well-managed diabetes and without diabetes (McIntosh et al., 2019). In any case, T2D is a risk factor for AD even after adjusting for vascular risk factors (Wang et al., 2012; Huang et al., 2014). Even if only 10% of diabetic patients end up suffering AD later in life, the number of patients with AD in the world will double (Ryu et al., 2019). Additionally, patients with AD have an increased risk of T2D, and up to 81% of patients with AD have T2D or impaired fasting glucose (Janson et al., 2004), supporting a two-way cross-talk between both pathologies.

Previous studies have shown that multiple players may underline the crosstalk between AD and T2D (Sims-Robinson et al., 2010; Ramos-Rodriguez et al., 2015; Infante-Garcia et al., 2016; Salas and De Strooper, 2019). Animal models have shown that metabolic alterations may affect and accelerate AD pathological features, ultimately contributing to cognitive impairment (Takeda et al., 2010; Ramos-Rodriguez et al., 2015; Infante-Garcia et al., 2016). In line with these observations, an ongoing project (Retinal and Cognitive Dysfunction in Type 2 Diabetes, recognized, Clinical Trials gov registration no. NCT04281186) funded by the European Commission (H2020 program-GA 847749) is currently investigating common mechanisms in the pathogenesis of diabetic retinopathy and cognitive impairment in the T2D population. Moreover, since AD has no successful treatment and brain insulin resistance may be significantly affected; clinical trials on insulin have been carried out. Regular insulin improves cognition in patients with mild cognitive impairment or mild to moderate AD, and is accompanied by improvements in tau-P181/Aβ42 ratio and specific brain volumes on MRI (Craft et al., 2017). Similarly, promising outcomes have been observed after intranasal insulin administration, such as preserved general cognition. Exploratory analyses revealed that changes in memory and function were associated to Aβ42 levels and tau/Aβ42 ratio in cerebrospinal fluid (Craft et al., 2012; Claxton et al., 2015). In line with these observations, patients with untreated diabetes present with higher phospho- and total-tau levels, higher phospho-tau/Aβ42 ratios, and higher rates of progression to dementia (McIntosh et al., 2019).

Other approaches have tried to improve cognitive function by targeting the activity of central glucagon-like peptide 1 (GLP-1) receptors (Hölscher and Li, 2010). Liraglutide (LRGT), specifically, is a GLP-1 receptor agonist that crosses the blood-brain barrier (Perry and Greig, 2005) and is currently being tested on patients with AD in the Evaluating Liraglutide in Alzheimer’s Disease (ELAD) clinical trial (NCT01843075) (Femminella et al., 2019). Studies that used *in vitro* and *in vivo* models show that LRGT improves AD pathological features, such as amyloid and tau pathologies, inflammation, or oxidative stress (McClean et al., 2011; Chen et al., 2017; Duarte et al., 2020; Jantrapirom et al., 2020), and cognitive alterations (Chen et al., 2017; Batista et al., 2018) in AD. However, to our knowledge, LRGT has not been tested on complex models that harbor both T2D and AD. We have analyzed the long-term effects of LRGT on APP/PS1xdb/db mice, a mixed murine model with severe brain complications derived from chronic T2D and AD. The APP/PS1xdb/db mice show brain atrophy and altered amyloid pathology when compared with APP/PS1 mice. Increased tau phosphorylation and spontaneous bleeding are also observed in the APP/PS1xdb/db mice. These complications result in early cognitive dysfunction that can be detected when T2D is established, but amyloid pathology is still scarce (Ramos-Rodriguez et al., 2015; Infante-Garcia et al., 2016). Altogether, this mixed murine model reproduces a more complex version of the pathology that includes classical neuropathological features of AD and T2D. LRGT treatment may ameliorate metabolic alterations and related vascular damage in diabetic mice while limiting amyloid and tau pathologies, inflammation, or neuronal damage associated with both AD and T2D. Therefore, by acting at different levels, LRGT may contribute in the maintenance of cognitive status when AD and T2D coexist, as commonly observed in the clinical arena (Janson et al., 2004).

## Materials and Methods

### Experimental Design: Animals and Treatments

AD-T2D mice were produced by cross-breeding APPswe/PS1dE9 (APP/PS1) [Tg (APPswe, PSEN1dE9) 85Dbo, stock no: 004462; Jackson Laboratories, Bar Harbor, ME, USA) with db/db mice (Harlan Laboratories, Boxmeer, Netherlands). APP/PS1 presents amyloid deposition at ≈4 months of age (Garcia-Alloza et al., 2006), and db/db mice are a functional knock out of the leptin receptor that only presents diabetic phenotype in homozygosis. Since heterozygous db/db mice do not present specific metabolic or central phenotype, the animals were grouped as follows: Control (APP/PS1^–/–^db/db^+/+^, APP/PS1^–/–^db/db^±^ mice), APP/PS1 (APP/PS1^±^db/db^+/+^ and APP/PS1^±^db/db^±^ mice), db/db (APP/PS1^–/–^db/db^–/–^ mice), and APP/PS1xdb/db (APP/PS1^±^db/db^–/–^ mice) as described previously (Ramos-Rodriguez et al., 2015; Infante-Garcia et al., 2016). The animals were randomly divided into groups and received LRGT as reported previously (Hansen et al., 2015). Briefly, the mice received an initial dose of subcutaneous LRGT (25 μg/kg/day) that was increased to 50, 100, 150, 200, 300, and 500 μg/kg/day daily during the first week From day seven, 500 μg/kg/day (133.3 nmol/Kg/day) of LRGT was administered to the mice daily for 20 weeks. T2D debuts at ≈6 weeks of age in db/db mice and by 26 weeks of age both AD and T2D are fully established in APP/PS1xdb/db mice. Therefore LRGT treatment commenced at 6 weeks of age an continued up to 26 weeks of age. Mice that did not receive LRGT received daily subcutaneous filtered PBS (vehicle). *In vivo* experiments included 31–34 females and 37–40 males randomly assigned to the treatment to complete the groups (Control *n* = 9–10, Control-LRGT *n* = 9–11, APP/PS1 *n* = 9–10, APP/PS1-LRGT *n* = 10–11, db/db *n* = 7–9, db/db-LRGT *n* = 8–12, APP/PS1xdb/db *n* = 5–7, and APP/PS1xdb/db-LRGT *n* = 7–8). Postmortem studies included 9–31 males and 11–26 females, depending on the assays, to complete the experimental groups (Control *n* = 3–8, Control-LRGT *n* = 3–6, APP/PS1 *n* = 4–8, APP/PS1-LRGT *n* = 4–9, db/db *n* = 3–7, db/db-LRGT *n* = 3–7, APP/PS1xdb/db *n* = 3–6, and APP/PS1xdb/db-LRGT *n* = 3–6). All experimental procedures were approved by the Animal Care and Use Committee of the University of Cadiz in accordance with the Guidelines for Care and Use of Experimental Animals (European Commission Directive 2010/63/UE and Spanish Royal Decree 53/2013).

### Metabolic Determination

Body weight, non-fasting blood glucose levels, and plasma insulin levels were determined before the commencement of the treatment and every 4 weeks until sacrifice at 26 weeks. Blood glucose levels were measured from nicked tails using the glucometer Optium Xceed (Abbott, London, United Kingdom). For plasma insulin determination, blood was collected from the tail vein and placed in tubes with potassium-EDTA (Sarstedt, Nümbrecht, Germany). Blood samples were centrifuged for 7 min, 6,500 rpm at 4°C, and plasma fraction was stored at –80°C until it was processed. Plasma insulin levels were measured using an ultrasensitive mouse enzyme-linked immunosorbent assay (ELISA) (Mercodia Inc., Winston-Salem, NC, USA).

### Morris Water Maze

Behavioral assessment commenced after 18 weeks of treatment with LRGT. The acquisition phase was assessed 12 days prior to sacrifice. The pool was a round tank of 90 cm in diameter and water temperature was 21 ± 2°C. The animals performed four trials/day for 4 days, with the platform submerged in quadrant 2. Swimming commenced in each of the four virtual quadrants that the pool was divided in. Time limit was 60 s/trial, with a 10-min intertrial interval. If an animal did not find the platform, it was placed on the platform for 10 s. The retention phase started 24 h after the acquisition phase was completed, and it consisted of a single trial with the platform removed. Time required to locate the platform in the acquisition phase, percentage of time spent in quadrant 2 during the retention phase, and swim speed were analyzed using the Smart software (Panlab, Barcelona, Spain).

### Actimetry and New Object Discrimination Task

Spontaneous motor activity test commenced the day after completion of the MWM. The mice were placed in a rectangular box (length 44 cm × width 22 cm × height 40 cm), and the distance traveled for 30 min was recorded. The new object discrimination test commenced the next day, as described previously (Dere et al., 2005; Ramos-Rodriguez et al., 2013). The animals were exposed to two objects, for habituation purposes, that were not used again during the object exploration task. On day 3, each mouse performed two sample trials and a test trial. On the first sample trial, the mice were allowed to explore for 5 min four copies of a novel object (navy balls) arranged in a triangle-shaped spatial configuration. After a 30-min delay, the mice performed a second sample trial with four novel objects (red cones) arranged in a quadratic-shaped spatial configuration, for 5 min. After 30 min, the mice performed a test trial with two copies of the object from sample trial 2 (“recent” objects) placed in the same position, and two copies of the object from sample trial 1 (“familiar” objects), with one placed in the same position (“old non-displaced” object) and the other in a new position (“familiar displaced” object). Integrated episodic memory for “what,” “where,” and “when” paradigms was analyzed as described previously (Dere et al., 2005). “What” was defined as the difference in time exploring familiar and recent objects, “where” was defined as the difference in time exploring displaced and non-displaced objects, and “when” was defined as the difference between time exploring familiar non-displaced objects and time exploring recent non-displaced objects.

### Rotarod

Motor coordination was assessed by the rotarod (Panlab Harvard Apparatus, Barcelona, Spain). An animal was placed on a horizontal rod (3 cm in diameter and 5.7 cm wide), which is rotated around its longitudinal axis, and the animal must walk forward to remain upright and not fall off (the height to fall is 16 cm). The animals were placed on the rod for 4 min at 4 revolutions per minute (rpm) for training purposes. During the test, the speed was increased from 4 to 40 rpm within 1 min. The time spent on the rod and the velocity when the animals fall was recorded.

### Tissue Processing and Cresyl Violet Staining

The animals were sacrificed by intraperitoneal pentobarbital overdose (120 mg/kg). Brains were immediately harvested and weighed. Right hemispheres were dissected and frozen at –80°C until they were used. Left hemispheres were fixed in PFA 4%, and 30-μm coronal sections were obtained with cryostat (Microm HM 525; Thermo Fisher Scientific, Madrid, Spain). Six sections located at 1.5, 0.5, –0.5, –1.5, –2.5, and –3.5 from Bregma, (Infante-Garcia et al., 2018), were selected. Briefly, the sections were dehydrated in 70% ethanol for 15 min and then incubated in a cresyl violet (Sigma, St. Louis, MO, USA) solution (0.5% w/v) for 10 min. After washing, tissue was fixed in 0.25% acetic acid in ethanol for 5 min, and subsequently in 100% ethanol and xylene for 2 min. The sections were mounted with DPX (Sigma, St. Louis, MO, USA). Images were acquired with an optical Olympus Bx60 microscope with an Olympus DP71 camera. Cell F (Olympus, Hamburg, Germany), Adobe Photoshop Elements and Image J software were used to process the images and measure cortex and hippocampus sizes.

### Prussian Blue Staining

Sections contiguous to those used for cresyl violet staining were incubated by Prussian blue iron staining and neutral red counterstaining as described previously (Desestret et al., 2009), to analyze spontaneous hemorrhages. Images were acquired with an Olympus Bx60 (Olympus, Tokyo, Japan) microscope with an Olympus DP71 camera (Olympus, Tokyo, Japan) to assess the complete cortex and hippocampus. The images were analyzed using the Image J software to quantify hemorrhage burden in the cortex and hippocampus.

### NeuN/DAPI Staining

Six sections located at 1.5, 0.5, –0.5, –1.5, –2.5, and –3.5 from Bregma were selected and blocked with BSA 3% and Triton X-100 0.5% during 1 h. Thereafter, the sections were incubated overnight at 4°C with anti-NeuN (Sigma, St. Louis, MO, USA) (Ref. MAB377) (1:200). Alexa Fluor donkey anti-mouse 594 was used as secondary antibody (Molecular Probes, Eugene, OR, USA) (Ref. A21203) (1:1,000), followed by DAPI 1 mg/ml (Sigma, St. Louis, MO, USA) (Ref. D9542) (1:3,000) counterstain for 1 h. Amyloid plaques were stained with thioflavin S (TS) (Sigma, St. Louis, MO, USA) (Ref. T1892) (0.01%) in H_2_O/ethanol (1:1) for 10 min. The sections were mounted and photographed using an Olympus Bx60 (Olympus, Tokyo, Japan) microscope with an Olympus DP71 (Olympus, Tokyo, Japan) camera. The percentage of NeuN-positive cells (normalized by total cells stained with DAPI) was quantified in the cortex, close (<50 μm from plaque border) and far (>50 μm) from amyloid plaques using the Image J software (Ramos-Rodriguez et al., 2016; Infante-Garcia et al., 2018).

### Axonal Immunostaining

Axonal curvature was analyzed after immunostaining with an SMI-312 antibody (Biolegend, San Diego, CA, USA) Ref. 837904) (1:1,000) as described (Infante-Garcia et al., 2017). Briefly, six sections 1 mm apart were pre-treated with hydrogen peroxide 3% and Triton X-100.5% for 20 min and blocked with 3% BSA for 1 h. Alexa Fluor goat anti-mouse 594 was used as secondary antibody (Molecular Probes, USA) (Ref. A11005) (1:200) for 1 h. Amyloid plaques were visualized with TS as described above. Micrographs of stained tissue were obtained with a Laser Olympus U-RFL-T (Olympus, Japan) fluorescent microscope and the MMIcellTools software. The Image J software was used for analysis purposes. Axon curvature ratio was calculated by dividing the end-to-end distance of a dendrite segment by the total length between the two segment ends. The distance to the closest amyloid plaque was measured at three points along each neurite, and the average distance was determined from these three measurements (Garcia-Alloza et al., 2010). At least 40 neurites were analyzed in each animal to complete 309–939 neurites/group. Curvatures were pooled in the proximity of amyloid plaques (up to 50 μm from the border), and neurites analyzed further from amyloid plaques borders were considered in SP-free areas. In the Control and db/db animals, axon curvature was compared with those measured in the APP/PS1 mice further than 50 μm from SP, as described previously (Garcia-Alloza et al., 2007a).

### Aβ and Microglia Immunostaining

PFA-fixed 30-μm sections were pre-treated with 70% formic acid for 10 min and blocked in 3% BSA and 0.5% Triton-X100 for 1 h. The sections were incubated with anti-Iba1 (Wako, Osaka, Japan) (Ref. 019-19741) (1:1,000) and 4G8 (Biolegend, London, United Kingdom) (Ref. 800702) (1:2,000), antibodies overnight at 4°C in 0.5% BSA followed by secondary antibodies Alexa Fluor donkey anti-rabbit 488 (Molecular Probes, Eugene, OR, USA) (Ref. A21206) and Alexa Fluor donkey anti-mouse 594 (Molecular Probes, Eugene, OR, USA) (Ref. A21203) (1:1,000) for 2 h. Images were acquired using a Laser Olympus U-RFL-T (Olympus, Tokyo, Japan) fluorescent microscope and the MMIcellTools software. Microglia burden (% covered area) in the cortex and hippocampus was also analyzed in the proximity of (<50 μm) and far (>50 μm) from amyloid plaques in the case of the APP/PS1 and APP/PS1xdb/db mice, and in random selected areas in mice without amyloid plaques (Control and db/db), as described previously (Garcia-Alloza et al., 2007b; Ramos-Rodriguez et al., 2015). The Image J software was used to analyze the number, size, and burden of Aβ deposits in the cortex and hippocampus.

### Aβ40, Aβ42, and Aβ Aggregates Enzyme-Linked Immunosorbent Assay

Soluble and insoluble Aβ40 and Aβ42 were quantified in the cortex and hippocampus with colorimetric ELISA kits (Wako, Osaka, Japan) (Aβ40, Ref. 294-62501; Aβ42, Ref. 290-62601) as described, with minor modifications (Infante-Garcia et al., 2017). Tissue (5–10 mg) was homogenized in 50 μl of Pierce^TM^ IP Lysis Buffer (Thermo Fisher Scientific, Madrid, Spain) (Ref. 87788) with Halt^TM^ (Thermo Fisher Scientific, Madrid, Spain) (Ref. 78440) phosphatase and protease inhibitor cocktail and centrifuged (14,500 rpm) for 12 min at 4°C. Soluble Aβ40 and Aβ42 levels were measured in supernatants. The resultant pellet was extracted with 50 μl of 70% formic acid and centrifuged at 14,500 rpm for 10 min. Insoluble fraction was neutralized and diluted 1:30 with 1M Tris (pH 11). Human Aβ40 and Aβ42 provided in the kit were used for standard curves. Aβ aggregates were quantified using Human Amyloid β (82E1-specific) Aβ Oligomers Assay Kit (IBL, Hamburg, Germany) (Ref. 27725). The cortex was homogenized (1/5 w/v) in Tris-buffered saline (TBS; 20 mM Tris-HCl, 140 mM NaCl, pH 7.5) containing Halt^TM^ phosphatase and protease inhibitor cocktail. Homogenates were then centrifuged (14,500 rpm) for 60 min at 4°C. The supernatant was collected and diluted 1:2 with EIA buffer provided in the kit. All absorbances were measured spectrophotometrically at 450 nm (MQX200R2; Biotek Instruments, Burlington, VT, USA), and data were expressed as pmol/g tissue.

### Total-Tau and Phospho-Tau Levels

Total tau and tau phosphorylation levels were measured in cortical and hippocampal samples as described previously (Infante-Garcia et al., 2017). Tissue was homogenized in Pierce^TM^ IP Lysis Buffer (Thermo Fisher Scientific, Madrid, Spain) (Ref. 87788) with Halt^TM^ (Thermo Fisher Scientific, Madrid, Spain) (Ref. 78440) phosphatase and protease inhibitor cocktail. The homogenates were sonicated and centrifuged at 4°C for 5 min at 15,000 g. Supernatants were collected, and protein concentration was determined by Bradford protein assay (Bio-Rad, Madrid, Spain) (Ref. 5000006). Proteins were separated on 10% acrylamide-bisacrylamide gels, followed by electrophoretic transfer to PVDF membranes (Bio-Rad, Madrid, Spain) (Ref. 1620177). Membranes were then immersed in blocking buffer (Thermo Fisher Scientific, Madrid, Spain) (Ref. WB7050) for 1 h and incubated overnight at 4°C with mouse anti-phospho-tau antibody (1:1,000) (clon AT8) Thermo Fisher Scientific, Agawan, MA, USA) (Ref. MN1020) (1:1,000). Membranes were washed and then incubated with Secondary Antibody Solution Alk-Phos. Conjugated (Anti-Mouse) (Thermo Fisher Scientific, Madrid, Spain) (Ref. 10013103), and a chemiluminescent inmunodetection system for mouse and rabbit primary antibodies (Invitrogen, Carlsbad, CA, USA). The membranes were washed, and signal was detected using Novex AP Chemiluminescent Substrate (Thermo Fisher Scientific, Madrid, Spain) (Ref. WP20002) in a ChemiDoc MP (Bio-rad, Madrid, Spain) imager. After stripping, the membranes were incubated with anti-total tau (1:1,000) (DAKO, Glostrup, Denmark) following the above procedure. Optical density was semi-quantified after normalizing to β-actin (Sigma, OR, USA) (Ref. A5441) (1:1,000) using the Image J software. Phospho-tau/total tau ratios were represented as percentage of control values.

### IR-A, IR-B, and IGF-1R mRNA Expression

For rt-qPCR analysis, RNA was isolated from the cortex using TRIzol^TM^ (Invitrogen, Carlsbad, CA, USA) (Ref. 15596026) following the instructions of the manufacturer and resuspended in purified nuclease-free water. The RNA was quantified using a BioTek Synergy^TM^ Mx (BioTek Instruments, Inc., Winooski, VT, USA) fluorometer. Complementary DNA (cDNA) was obtained from 500-ng RNA using iScript^TM^ cDNA Synthesis Kit (Bio-Rad Laboratories Inc., Hercules, CA, USA) (Ref.1708890) on Techne Genius Thermal Cycler (Techne Ltd., Cambridge, United Kingdom). The 15-μl RT-qPCR reaction mix contained 7.5 μl 2X iTaq^TM^ Universal SYBR^®^ Green Supermix (Bio-Rad Laboratories Inc., Hercules, CA, USA) (Ref. 1725122) 200 nmol (for IR-varA, IR-varB, and rRNA18S) or 900 nmol (for IGF-I) of forward and reverse primers, and 1 μl of the sample. The PCR thermal profile included 40 cycles of denaturation at 95°C for 10 s, annealing at temperature according to each set of primers (61°C for IR-varA, 59°C for IR-varB, 64°C for IGF-I, and 55°C for rRNA18S) for 15 s, and extension at 72°C for 20 s, followed by melting curve analysis. Each sample was analyzed in triplicate, and 6–9 mice per group were included in the study. The mRNA level of rRNA18S was used as internal control. Relative quantification values of mRNA expression were calculated as 2–ΔΔCt with the comparative Ct method. First, internal control Ct values were subtracted from the gene-of-interest Ct values to derive a ΔCT value. The relative expression of the gene of interest was then evaluated using the expression 2−ΔΔCt, where the value for ΔΔCt was obtained by subtracting the ΔCt of the calibrator from each ΔCT, using the mean of the control (Control animals) as the calibrator. The oligonucleotide primers used in this study were designed by BLAST and were obtained from Merck KGaA (Madrid, Spain) for IR-varA (FW:TTTGTCCCCAGGCCATCC-RV:ATCTGGAAGTGTGAGTGTGG), IR-varB (FW:AATGGTG CCGAGGACAGTA-RV:ATCTGGAAGTGTGAGTGTGG) and IGF-I (FW:CACAACTACTGCTCCAAAGACAAA-RV:TTTTC CGTCACCTCCTCCAC); rRNA18S (FW:CTCAACACGGGAA ACCTCAC-RV:CTCAACACGGGAAACCTCAC).

### Statistical Analysis

One-way ANOVA for independent samples, followed by Tukey’s b or Tamhane test, was performed when all the eight groups under study were compared. One-way ANOVA was also performed when only treated and untreated APP/PS1 and APP/PS1xdb/db mice were analyzed (Aβ levels, amyloid plaques, microglia, and neuronal curvature close to amyloid plaques). Two-way ANOVA (groupXday) was performed to analyze the acquisition phase in the MWM test. The SPSS v.24 software was used for all the statistical analyses. One-way ANOVA for independent samples was performed for further analysis of individual days during acquisition in the MWM. Data are presented as mean ± SEM.

## Results

### Liraglutide Reduces Metabolic Alterations in T2D and AD-T2D Mice

**FIGURE 1:**
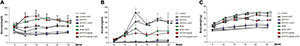
Long-term liraglutide (LRTG) treatment ameliorates metabolic alterations in T2D and AD-T2D mice. **(A)** Non-fasting plasma glucose, **(B)** insulin, and **(C)** body weight were measured every 4 weeks in controls, APP/PS1, db/db and APP/PS1xdb/db mice treated with vehicle or LGRT 500 μg/kg for 20 weeks (from weeks 6 to 26) (complete statistical analysis included as a Supplementary Material). **(A)** LRGT significantly reduced postprandial glucose levels in diabetic mice: (^##^*p* < 0.01 Control, Control-LRGT, APP/PS1, APP/PS1-LRGT; 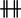
*p* < 0.01 vs. Control; ^††^*p* < 0.01 Control, Control-LRGT, APP/PS1, APP/PS1-LRGT, db/db-LRGT, and APP/PS1xdb/db-LRGT; 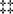
*p* < 0.001 vs. Control, Control-LRGT, APP/PS1, APP/PS1-LRGT, and db/db-LRGT; 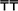
*p* < 0.01 vs. Control-LRGT). **(B)** Insulin levels were maintained by long-term LRGT treatment. At 6 weeks of age, immediately before the commencement of LRGT treatment, insulin levels were significantly increased in APP/PS1xdb/db mice (^##^*p* < 0.01 Control, Control-LRGT, APP/PS1, APP/PS1-LRGT; 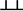
*p* < 0.01 vs. Control, Control-LRGT, APP/PS1, APP/PS1-LRGT, db/db, and APP/PS1xdb/db; 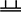
*p* < 0.01 vs. Control, Control-LRGT, APP/PS1, APP/PS1-LRGT, and APP/PS1xdb/db). **(C)** LRGT maintained body weight in APP/PS1-LRGT (^##^*p* < 0.01 vs. Control, Control-LRGT, APP/PS1, and APP/PS1-LRGT; ^##^*p* < 0.01 vs. Control, Control-LRGT, APP/PS1, and APP/PS1-LRGT; 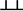
*p* < 0.01 vs. Control, Control-LRGT, APP/PS1, APP/PS1-LRGT, and APP/PS1xdb/db). Data are representative of 5–12 animals, and differences were detected by one-way ANOVA followed by Tukey’s b or Tamhane test.

When we analyzed glucose level evolution along treatment, we did not detect a significant groupXweek effect by two-way ANOVA for independent samples [*F*_(35, 380)_ = 1.18, *p* = 0.227]. However, further assessment of individual days revealed that the LRGT treatment reduced glucose levels in diabetic mice (Figure 1A). On week 6, in baseline untreated animals, plasma glucose in the db/db mice and APP/PS1xdb/db crosses were significantly increased compared with the Control and APP/PS1 mice. This increase in non-fasting glucose levels was more severe in the APP/PS1xdb/db animals (*p* < 0.001). By 10 weeks of age, glucose levels were significantly higher in the untreated db/db and APP/PS1xdb/db mice when compared with the LRGT-treated and untreated Control and APP/PS1xdb/db mice (*p* < 0.01). Differences in glucose levels between the untreated diabetic (db/db and APP/PS1xdb/db mice) and non-diabetic mice were maintained up to week 26 (*p* < 0.01). The LRGT treatment helped in limiting hyperglucemia in the db/db mice, and glucose levels reached control values by week 10 (after 4 weeks of LRGT treatment). Similarly, LRGT reduced non-fasting glucose levels in the APP/PS1xdb/db mice when compared with the untreated APP/SP1xdb/db animals by 10 weeks of age, and a similar profile was observed by 14 weeks of age. By 18 weeks, glucose levels in the APP/PS1xdb/db-LRGT mice were similar to those detected in the Control and APP/PS1 animals. Glycemic control was maintained in the db/db-LRGT and APP/PS1xdb/db-LRGT mice until the end of treatment (26 weeks of age).

When we analyzed insulin levels, we detected a significant groupXweek effect by two-way ANOVA for independent samples along treatment [*F*_(35, 384)_ = 2.64, *p* < 0.01]. Further differences were observed among the groups when we analyzed individual weeks (Figure 1B). On week 6, basal insulin levels in untreated animals were increased in the db/db mice, although differences with the Control and APP/PS1 animals only reached statistical significance in the APP/PS1xdb/db mice (*p* < 0.01). The fact that insulin levels were significantly increased in the APP/PS1xdb/db mice suggests an earlier metabolic compromise in the crossed model that requires an increase in pancreatic activity. By 10 weeks of age, statistical differences were observed in the db/db-LRGT and APP/PSxdb/db-LRGT mice when compared with the non-diabetic animals (Control and APP/PS1), suggesting an increase in insulin production in the LRGT-treated mice that allows for better glycemic control (*p* < 0.01). This situation was maintained from 14 to 26 weeks of age, and LRGT helped to increase insulin levels in the diabetic mice (db/db-LRGT and APP/PS1xb/db-LRGT) to control hyperglycemia.

We also detected a significant groupXweek effect by two-way ANOVA for independent samples [*F*_(35, 400)_ = 3.13, ^∗∗^*p* < 0.01] when we analyzed body weight. Further assessment of individual weeks revealed that the LRGT treatment helped maintain body weight in cachectic APP/PS1xdb/db mice, as shown previously (Infante-Garcia et al., 2018; Hierro-Bujalance et al., 2020) (Figure 1C). By week 6 at baseline, the untreated diabetic mice (db/db and APP/PS1xdb/db) were overweight when compared with the non-diabetic mice (Control and APP/PS1) (*p* < 0.01). By week 10, all the diabetic mice, treated and untreated, presented significantly higher body weight when compared with the Control and APP/PS1 animals (*p* < 0.01), and these differences were maintained up to week 18 (*p* < 0.01). By week 22, the untreated APP/PS1xdb/db mice were still overweight when compared with the Control and APP/PS1 animals (*p* < 0.01); however, a slight reduction in body weight could be detected, indicative of a cachectic state, as described previously (Infante-Garcia et al., 2018; Hierro-Bujalance et al., 2020). Nevertheless, LRGT helped in maintaining the body weight of the APP/PS1xd/db mice, avoiding weight loss from week 22 until the end of the study (26 weeks).

### Liraglutide Improves Cognitive Impairment in APP/PS1xdb/db Mice

**FIGURE 2:**
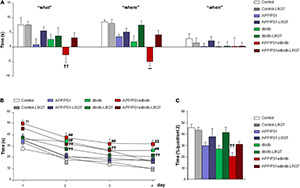
LRGT treatment reduced cognitive impairment in APP/PS1xdb/db mice. Control, APP/PS1, db/db, and APP/PS1xdb/db animals were analyzed in the new object discrimination test for “what,” “when,” and “where” paradigms **(A)** as well as in the **(B,C)** Morris water maze test. Behavioral assessment commenced on week 24, after 18 weeks of LRGT treatment (500 μg/kg/day), and was completed on week 26 (complete statistical analysis included as a Supplementary Material). **(A)** LRGT improved episodic memory in the new object discrimination test. No differences were observed for the “when” paradigm; however, significant improvement was observed for the “what” (^††^*p* = 0.009 vs. Control, Control-LRGT, and APP/PS1-LRGT) and “where” (**p < 0.001 vs. rest of the groups) paradigms. **(B)** LRGT also improved the performance along the acquisition phase in the MWM (^††^*p* < 0.01 vs. Control, Control-LRGT, APP/PS1, APP/PS1-LRGT, db/db, and db/db-LRGT; ^##^*p* < 0.01 vs. Control, Control-LRGT, APP/PS1, and APP/PS1-LRGT; 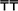
*p* < 0.01 vs. Control and Control-LRGT; 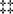
*p* < 0.01 vs. Control, Control-LRGT, APP/PS1, APP/PS1-LRGT, db/db-LRGT, and APP/PS1xdb/db-LRGT). **(C)** In the retention of the MWM, we observed that LRGT treatment also improved the performance of APP/PS1xdb/db-LRGT mice (^††^*p* = 0.002 vs. Control, Control-LRGT, and db/db-LRGT) (complete statistical analysis included in Supplementary Material). *Data are representative of 5–12 animals*, and differences were detected by one-way ANOVA followed by Tukey’s b or Tamhane test.

As described previously, episodic memory was affected in the APP/PS1, db/db, and APP/PS1xdb/db mice in the new object discrimination test (Ramos-Rodriguez et al., 2015; Infante-Garcia et al., 2016), reaching statistical significance in the case of “what” and “where” paradigms when all the groups under study were compared (Figure 2A). Differences reached statistical significance when the untreated APP/PS1xdb/db mice were compared in the “what” and “where” paradigms (*p* < 0.01). Importantly, the LRGT treatment counterbalanced this situation, and the APP/PS1xdb/db-LRGT mice performed like the Control mice in both paradigms.

We did not detect a significant groupXday effect by two-way ANOVA for independent samples [*F*_(21, 1113)_ = 0.985, *p* = 0.479] when spatial memory was analyzed in the acquisition phase of the MWM. However, individual day assessment revealed that cognitive impairment in the db/db and APP/PS1xdb/db mice was significantly ameliorated by the LRGT treatment (Figure 2B). On day 1 of the acquisition phase, the APP/PS1xdb/db mice were already compromised when compared with the Control, Control-LRGT, APP/PS1, APP/PS1-LRGT, db/db, and db/db-LRGT mice (*p* < 0.01). This compromise was also observed on acquisition days 2–4 for the naïve APP/PS1xdb/db mice and db/db animals when compared with the LRGT-treated and untreated non-diabetic mice (Control and APP/PS1). An improvement was observed in diabetic mice after the LRGT treatment, although the times to locate the platform were still longer than those observed in the Control and Control-LRGT mice (Figure 2B). In the retention phase of the MWM, we observed that the APP/PS1xdb/db mice were compromised and spent significantly shorter times in the quadrant where the platform used to be located when compared with the Control mice (*p* = 0.02). However, after the LRGT treatment, differences were no longer observed between the APP/PS1xdb/db-LRGT and Control mice (Figure 2C).

We also analyzed motor activity by assessing different paradigms. When we analyzed the distance traveled in the open field, we observed that the diabetic mice (db/db and APP/PS1xdb/db) traveled shorter distances than the non-diabetic animals, although no statistical differences were observed among the groups under study (Table 1). Swimming speed in the MWM was also used to characterize motor alterations in all the groups under study (Table 1). As observed previously, in the diabetic mice (db/db and APP/PS1xd/db mice), swimming speeds were lower than those detected in the Control and APP/PS1 animals (*p* < 0.01). Similar differences between diabetic and non-diabetic mice were observed when motor activity was analyzed by time (*p* < 0.01) or maximum speed (*p* < 0.01) in the rotarod test (Table 1). However, no differences were observed in any of the paradigms (distance traveled in the open field, swimming speed in the MWR, and rotarod) when the db/db-LRGT or the APP/PS1xdb/db-LRGT mice were compared with the untreated db/db or the APP/PS1xdb/db animals, suggesting that the observed improvement in learning and memory is not due to changes in motor activity.

**TABLE 1:** Motor activity assessment on liraglutide (LRGT)-treated mice.

	**Distance traveled spontaneous motor activity (cm)**	**Swimming speed (cm/s)**	**Time in rotarod (s)**	**Rotarod speed (rpm)**
Control	11921.50 ± 378.18	22.09 ± 1.04	15.55 ± 2.42	16.36 ± 2.18
Control-LRGT	11815.75 ± 658.06	19.33 ± 1.27	11.92 ± 2.11	13.25 ± 1.31
APP/PS1	12715.31 ± 707.07	21.28 ± 1.39	18.00 ± 2.74	15.80 ± 1.79
APP/PS1-LRGT	11456.10 ± 548.60	19.83 ± 0.71	14.30 ± 2.03	13.80 ± 1.31
db/db	9446.47 ± 1105.73	14.56 ± 0.88^‡‡^	2.33 ± 0.88^‡‡^	6.11 ± 0.80^‡‡^
db/db-LRGT	9343.74 ± 789.27	13.23 ± 0.59^‡‡^	2.44 ± 0.84^‡‡^	5.88 ± 0.63^‡‡^
APP/PS1xdb/db	9814.83 ± 2189.93	14.07 ± 1.48^‡‡^	2.67 ± 1.31^‡‡^	5.50 ± 0.92^‡‡^
APP/PS1xdb/db-LRGT	9626.63 ± 1344.51	14.48 ± 1.36^‡‡^	2.00 ± 0.38^‡‡^	5.75 ± 0.31^‡‡^

*No differences were observed when the distance traveled by all the groups under study in the spontaneous motor activity test was compared [F_(6, 67)_ = 2.1, p = 0.046, no further differences detected]. Swimming speed in the MWM was significantly reduced in diabetic mice, and no differences were observed after LRGT treatment [F_(6, 61)_ = 9.2, ^‡‡^p < 0.01 vs. Control, Control-LRGT, APP/PS1, and APP/PS1-LRGT]. A similar profile was observed in the rotarod test when we analyzed time [F_(6, 68)_ = 11.73, ^‡‡^p < 0.01 vs. Control, Control-LRGT, APP/PS1, and APP/PS1-LRGT] and maximum speed [F_(6, 67)_ = 11.31, ^‡‡^p < 0.01 vs. Control, Control-LRGT, APP/PS1 and APP/PS1-LRGT]. Data are representative of 5–14 animals, and differences were detected by one-way ANOVA followed by Tukey’s b test or Tamhane as required.*

### Liraglutide Limits Brain Atrophy and Neuronal Loss

**FIGURE 3:**
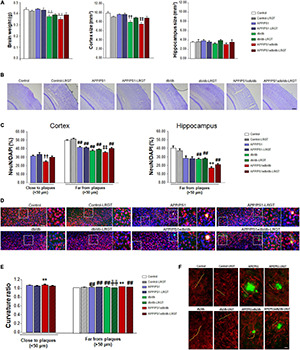
Brain atrophy, neuronal density, and curvature are reduced by LRGT treatment. **(A,B)** Brain weight, cortex and hippocampal size, **(C,D)** NeuN/DAPI ratio, and **(E,F)** axonal curvature ratio were analyzed in all four genotypes (Control, APP/PS1, db/db, and APP/PS1xdb/db) under study and compared with animals by week 26, after 20 weeks on LRGT treatment (500 μg/kg/day) (complete statistical analysis included as a Supplementary Material). **(A)** Long-term LRGT limited brain weight loss (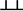
*p* < 0.01 vs. Control, Control-LRGT, APP/PS1, APP/PS1-LRGT, db/db, and APP/PS1xdb/db). Cortical size was significantly improved by the LRGT treatment (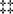
*p* < 0.01 vs. Control, Control-LRGT, APP/PS1, APP/PS1-LRGT, db/db-LRGT, and APP/PS1xdb/db-LRGT; ^††^*p* < 0.01 vs. Control, Control-LRGT, APP/PS1, and APP/PS1-LRGT). No differences were observed in the hippocampus. Data are representative of 4–5 animals. **(B)** Illustrative example of cresyl violet staining showing reduced cortical size in db/db and APP/PS1xdb/db mice. Scale bar = 200 um. **(C)** Neuronal density was reduced in the proximity of amyloid plaques in APP/PS1xdb/db mice, and LRGT ameliorated this situation (^††^*p* = 0.008 vs. APP/PS1 and APP/PS1-LRGT). A similar profile is observed in cortical and hippocampal areas with no amyloid plaques (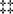
*p* < 0.001 vs. Control, Control-LRGT, APP/PS1, APP/PS1-LRGT, db/db-LRGT, and APP/PS1xdb/db-LRGT; ^##^*p* < 0.01 vs. Control and Control-LRGT; + + *p* < 0.01 vs. Control, Control-LRGT, APP/PS1, APP/PS1-LRGT, db/db, and db/db-LRGT). Data are representative of five animals. **(D)** Illustrative example of NeuN (red) and DAPI (blue) staining in areas located in the proximity of amyloid plaques (TS staining, green) and in areas without amyloid plaques. Zoom-in images of representative regions are marked by white squares and presented next to the original image, including areas with and without amyloid plaques. Scale bar = 50 μm, insets scale bar = 25 μm. **(E)** LRGT reduced curvature ratio in the proximity of amyloid plaques (**p = 0.004 vs. rest of the groups) and in areas free of amyloid plaques (**p < 0.01 vs. rest of the groups, ^##^*p* < 0.01 vs. Control and Control-LRGT, 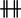
*p* < 0.01. vs. Control) (complete statistical analysis included in Supplementary Material). Data are representative of five animals per group (308–920 neurons/group). **(F)** Illustrative examples of SMI-312 (red) and TS (green) staining in the proximity of and far from amyloid plaques (yellow lines mark representative neurites). Scale bar = 10 μm.

Severe brain atrophy was detected in the db/db and APP/PS1xdb/db mice as described previously (Ramos-Rodriguez et al., 2015; Infante-Garcia et al., 2016) when all the groups under study were compared (Figure 3A). Brain weight was comparable between controls and APP/PS1 mice receiving vehicle, whereas the brain weight of vehicle-treated db/db and APP/PS1xdb/db mice was significantly reduced. On the other hand, LGRT-treated db/db and APP/PS1xdb/db brain weight was comparable with that of the vehicle and LGRT-treated controls and APP/PS1 mice (Figure 3A). Further assessment of brain structures revealed that cortical size was significantly reduced in the db/db and APP/PSxdb/db mice when compared with the untreated Control and APP/PS1xdb/db animals (*p* < 0.01). Nevertheless, the LRGT treatment significantly improved this situation in the diabetic animals, and cortical size in db/db-LRGT and APP/PS1xd/db-LRGT was comparable with that of the vehicle- and LRGT-treated controls and APP/PS1 mice (Figures 3A,B). Differences did not reach statistical significance in the hippocampus (Figure 3A).

We observed that neuronal density in the cortex was significantly reduced in the proximity of amyloid plaques when the APP/PS1xdb/db mice were compared with the APP/PS1 and APP/PS1-LRGT treated animals, while the LRGT-treated APP/PS1xdb/db mice presented values similar to those detected in the APP/PS1 and APP/PS1-LRGT mice (Figures 3C,D). In cortical areas far from amyloid plaques, we detected a significant compromise in the untreated APP/PS1 and db/db mice when compared with the Control and Control-LRGT mice (*p* < 0.01). This effect was more severe in the untreated APP/PS1xdb/db mice, and the LRGT treatment contributed to improve NeuN/DAPI ratio in this group, reaching statistical significance when the APP/PS1xdb/db and APP/PS1xdb/db-LRGT mice were compared (Figures 3C,D). The number of plaques in the hippocampus was low at 6 months of age, and in areas far from amyloid plaques, we observed that LRGT also helped in maintaining neuronal density (Figure 3C). In the untreated db/db mice, we observed that NeuN/DAPI ratios were significantly lower than those measured in the LRGT-treated and untreated Control and APP/PS1 animals (*p* < 0.01). The APP/PS1xdb/db mice showed a more severe reduction in NeuN/DAPI ratio that was statistically significant when compared with the LRGT-treated and untreated Control, APP/PS1, and db/db animals. The LRGT treatment improved NeuN/DAPI ratios in the hippocampus of the APP/PS1xdb/db mice, although they did not reach Control or APP/PS1 values (Figure 3C).

### Neuronal Curvature Is Reduced After Liraglutide Treatment

Curvature ratio was increased in the proximity of amyloid plaques when the untreated APP/PS1xdb/db animals were compared with the APP/PS1 and APP/PS1-LRGT mice (*p* = 0.04). On the other hand, the LRGT treatment significantly improved neuronal curvature ratio in the APP/PS1xdb/db mice when compared with the untreated APP/PS1xdb/db mice (*p* = 0.004) (Figures 3E,F). A similar profile was observed in areas free from amyloid plaques when all the groups under study were compared (Figures 3E,F), and a significant compromise was observed in the untreated APP/PS1xdb/db animals when compared with the rest of the groups (*p* < 0.01). The LRGT treatment helped in limiting this situation, and while neuronal curvature in the APP/PS1xdb/db-LRGT mice did not reach Control values, a significant straightening effect was observed when the treated APP/PS1xdb/db mice where compared with the naïve animals (*p* < 0.01) (Figure 3E).

### Liraglutide Reduces Aβ Pathology

**FIGURE 4:**
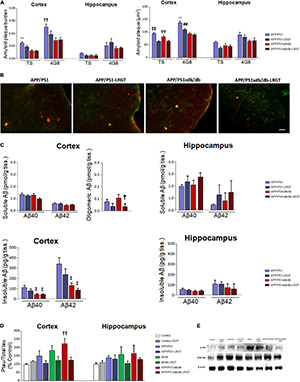
LRGT affects amyloid and tau pathologies in APP/PS1xdb/db mice. **(A,B)** Amyloid plaque burden and size are quantified by thioflavin S and 4G8 staining. **(C)** Aβ40, Aβ42, and Aβ aggregates are also determined in APP/PS1 and APP/PS1xdb/db mice after liraglutide treatment (500 μg/kg/day) for 22 weeks. **(D,E)** Phospho-tau/total tau ratios are also measured in all the genotypes (Control, APP/PS1, db/db, and APP/PS1xdb/db) under study untreated or after LRGT treatment (complete statistical analysis included as a Supplementary Material). **(A)** LRGT treatment reduced amyloid plaque burden in the cortex from APP/PS1 mice (**p < 0.01 vs. rest of the groups; ^††^*p* < 0.001 vs. APP/PS1xdb/db, and APP/PS1xdb/db-LRGT). No differences were observed in the hippocampus. LRGT treatment also reduced amyloid plaque size in the cortex from APP/PS1xdb/db mice (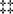
*p* < 0.01 vs. APP/PS1-LRGT and APP/PS1xdb/db-LRGT, 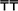
*p* < 0.001 vs. APP/PS1-LRGT; **p < 0.01 vs. rest of the groups, **^##^***p* < 0.001 APP/PS1xdb/db-LRGT). No differences were observed in the hippocampus. Data are representative of five animals. **(B)** Illustrative image of TS (green) and 4G8 (red) staining of amyloid plaques in the cortex from all the groups studied. Scale bar = 50 μm. **(C)** No differences were observed when soluble Aβ40 or Aβ42 levels were analyzed in the cortex. However, Aβ aggregates were significantly reduced in APP/PS1xdb/db mice on LRGT treatment (†*p* = 0.046 vs. APP/PS1xdb/db). No statistical differences were observed in the hippocampus for soluble Aβ40 or Aβ42 levels. Insoluble Aβ40 (‡*p* = 0.026 vs. APP/PS1) and Aβ42 (‡*p* = 0.011 vs. APP/PS1) levels are reduced in APP/PS1xdb/db when compared with APP/PS1 animals, and LRGT treatment contributes to further reductions in the cortex. No differences were observed in the hippocampus for insoluble Aβ40 or Aβ42. Data are representative of 6–9 animals. **(D)** Tau phosphorylation was reduced in the cortex after LRGT treatment (^††^*p* = 0.009 vs. Control). Differences did not reach statistical significance in the hippocampus. Data are representative of 3–11 mice. **(E)** Illustrative example of Western blot for phospho-tau, total tau, and β-actin in the cortex from all the groups studied.

As described previously, amyloid plaque burden is lower in the cortex from the APP/PS1xdb/db mice when compared with the APP/PS1 animals (Infante-Garcia et al., 2016). TS staining showed that amyloid plaque burden was significantly higher in the APP/PS1 mice than in the APP/PS1xdb/db animals (*p* < 0.001). The LRGT treatment contributed in reducing amyloid plaque burden in the APP/PS1 animals (*p* < 0.001), whereas differences did not reach statistical significance when the untreated APP/PS1xdb/db mice were compared with the APP/PS1xdb/db-LRGT mice (Figures 4A,B). A similar profile was observed after 4G8 immunostaining, and differences reached statistical significance when the APP/PS1 mice were compared with the APP/PS1xdb/db and APP/PS1xdb/db-LRGT mice. No differences were observed among the groups when we assessed amyloid plaque burden in the hippocampus (Figure 3A). TS staining also revealed that the LRGT treatment reduced amyloid plaque size in the cortex from the APP/PS1 and APP/S1xdb/db mice when compared with the untreated animals (*p* < 0.01) (Figure 4A). We also observed that the LRGT treatment reduced 4G8-labeled plaques in the APP/PS1 and APP/PSxdb/db mice (Figure 4A). Amyloid plaque size was not significantly affected in the hippocampus when the treated and untreated APP/PS1 and APP/PS1xdb/db mice were compared (Figure 4A).

Soluble Aβ is slightly favored in the APP/PS1xdb/db mice when compared with the APP/PS1 mice (Ramos-Rodriguez et al., 2015). While we observed this profile, differences did not reach statistical significance when the LRGT-treated and untreated APP/PS1 and APP/PS1xdb/db mice were compared. Similar outcomes were detected when soluble Aβ42 levels were analyzed (Figure 4C). The LRGT treatment significantly reduced Aβ aggregates in the APP/PS1xdb/db mice when compared with the untreated animals (*p* = 0.046) (Figure 4C). Soluble Aβ42 levels were not affected in the hippocampus when all the four groups (APP/PS1, APP/PS-LRGT, APP/PS1xdb/db, and APP/PS1xdb/db-LRGT) were compared (Figure 4C). Insoluble Aβ40 (*p* = 0.026) and Aβ42 (*p* = 0.011) levels were reduced in untreated APP/PS1xdb/db when compared with the untreated APP/PS1 animals. Differences did not reach statistical significance when the APP/PS1xdb/db mice were compared with the APP/PS1xdb/db-LRGT animals (Figure 4C). Differences did not reach statistical significance when insoluble Aβ40 or Aβ42 levels were compared in the hippocampus (Figure 4C).

### Liraglutide Limits Tau Pathology

We observed an increase in cortical tau phosphorylation in the db/db animals, although differences only reached statistical significance when the untreated APP/PS1xdb/db mice were compared with the Control animals (*p* = 0.009). LRGT reduced phospho-tau/total tau ratio in the APP/PS1xdb/db mice, and differences were no longer observed when the APP/PS1xdb/db-LRGT mice were compared with the Control animals (Figures 4D,E). Likewise, we also detected an increase in hippocampal tau phosphorylation in the db/db and APP/PS1xdb/db mice. While the LRGT treatment seemed to counterbalance this situation, no statistical differences were detected among any of the groups under study (Figure 4D).

### Liraglutide Reduces Spontaneous Bleeding in APP/PS1xdb/db Mice

**FIGURE 5:**
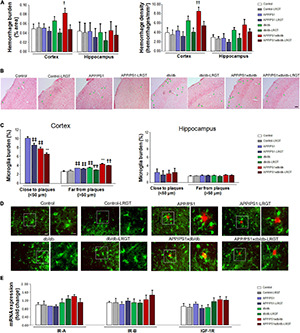
Spontaneous bleeding and inflammation are reduced after LRGT treatment while IR-A, IR-B, and IGF-1R mRNA expression is not affected. Prussian blue staining is used to quantify hemorrhage burden and density in the cortex from untreated and treated mice (LRGT, 500 μg/kg/day) **(A,B)**. Microglia burden was quantified by Iba1 immunostaining in the proximity of (<50 μm) and far (>50 μm) from amyloid plaques in untreated and LRGT-treated animals **(C,D)**. IR-A, IR-B, and IGF-1R mRNA expression is also determined in the cortex from untreated and LRGT-treated mice **(E)** (complete statistical analysis included as a Supplementary Material). **(A)** LRGT treatment reduces hemorrhage burden in the cortex (^†^*p* = 0.012 vs. Control, Control-LRGT, APP/PS1, APP/PS1-LRGT, db/db-LRGT, and APP/PS1xdb/db-LRGT). A similar profile was observed when we analyzed cortical hemorrhage density (^††^*p* < 0.001 vs. Control, Control-LRGT, APP/PS1, db/db-LRGT, and APP/PS1xdb/db-LRGT). No differences were detected in the hippocampus when hemorrhage burden or density was analyzed. Data are representative of 3–5 mice (489–1,012 hemorrhages/group). **(B)** Illustrative example of cortical hemorrhages stained with Prussian blue. Green arrows point at individual hemorrhages. Scale bar = 100 μm. **(C)** LRGT treatment reduced cortical microglia burden in APP/PS1 and APP/PS1xdb/db mice, in the proximity of amyloid plaques (**p < 0.01 vs. rest of the groups, 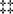
*p* < 0.01 vs. APP/PS1). LRGT also reduced microglia burden in cortical amyloid plaque-free areas in diabetic mice (**p < 0.01 vs. rest of the groups, ^††^*p* < 0.01 vs. Control, Control-LRGT, APP/PS1, APP/PS1-LRGT, db/db, and db/db-LRGT, 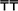
*p* < 0.01 vs. Control, Control-LRGT, APP/PS1, APP/PS1-LRGT, and db/db, 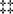
*p* < 0.01 vs. Control and Control-LRGT). No statistical differences were observed in the hippocampus close or far from amyloid plaques. Data are representative of five mice (cortex 572–748 ROIs/group; hippocampus 108–230, ROIs/group). **(D)** Illustrative example of cortical immunostaining for Iba1 (microglia, green) and 4G8 (amyloid plaques, red). Scale = 100 μm. Zoom-in images of representative regions are marked by white squares and presented next to the original image. Scale bar = 50 μm, inset scale bar = 10 μm. **(E)** No differences were observed in the cortex when we analyzed IR-A, IR-B, or IGF-1R mRNA expression. Data are representative of 6–9 mice.

As described previously, hemorrhage burden in the cortex was significantly increased in the db/db and APP/PS1xdb/db mice when compared with the naïve Control and APP/PS1 animals (*p* = 0.012). Interestingly, the LRGT treatment reduced hemorrhage burden in the cortex from the db/db and APP/PS1xdb/db mice when compared with untreated animals of these genotypes. Cortical hemorrhage density was also significantly higher in the db/db and APP/PS1xdb/db mice than in the Control and APP/SP1 animals, and LRGT successfully limited hemorrhage density in the diabetic animals (*p* < 0.001) (Figures 5A,B). We did not observe significant differences among the groups when hemorrhage burden and density were analyzed in the hippocampus (Figure 5A).

### Liraglutide Limits Microglia Activation in APP/PS1xdb/db Mice

Cortical microglia burden was significantly lower in the close proximity of amyloid plaques in APP/PS1xdb/db mice when compared with the APP/PS1 mice (*p* < 0.001), and the LRGT treatment reduced microglia burden both in the APP/PS1 and APP/PS1xdb/db mice when compared with the untreated APP/PS1 and APP/PS1xdb/db groups (Figures 5C,D). On the other hand, microglia burden was significantly higher in the untreated APP/PS1xdb/db mice than in the untreated Control, APP/PS1, and db/db mice in areas with no amyloid plaques (*p* < 0.001). The LRGT treatment reduced microglia burden in the db/db mice, and more robust differences were observed in the APP/PS1xdb/db mice after the LRGT treatment (*p* < 0.001). Differences did not reach statistical significance in the hippocampus when microglia burden in the proximity or far from the amyloid plaques (Figure 5C) was analyzed.

### Liraglutide Has No Effect on mRNA Expression of IR-A, IR-B, or IGF-1R

When we analyzed IR-A mRNA expression, we did not observe any differences in the cortex from any of the groups under study (Figure 5E). Similar outcomes were observed for IR-B or IGF-1R (Figure 5E).

## Discussion

Patients with AD are in tremendous need of new therapeutic opportunities (Arvanitakis et al., 2019). The close relationship between T2D and AD (Ryu et al., 2019) supports the study on antidiabetic agents slowing down or counterbalancing AD brain pathology and cognitive impairment in animal models (Infante-Garcia et al., 2018; Hierro-Bujalance et al., 2020) and patients (Cao et al., 2018). GLP-1 analogs reduce amyloid and tau pathologies, inflammation, and cognitive impairment in different models (McClean et al., 2011; Chen et al., 2017; Batista et al., 2018; Duarte et al., 2020; Jantrapirom et al., 2020). Also, preliminary studies on humans show that LRGT might be neuroprotective in individuals at risk of AD, improving intrinsic connectivity within default mode network in the brain (Watson et al., 2019). Moreover, the ELAD trial is currently assessing the effect of LRGT on patients with AD (Femminella et al., 2019). However, as far as we know, no studies have analyzed the role of LRGT in complex models that harbor both AD and T2D, as regularly seen in the clinical arena. Therefore, we have analyzed the effects of long-term LRGT treatment in APP/PS1xdb/db mice, a mixed murine model that reproduces severe brain complications associated with T2D and AD (Infante-Garcia et al., 2016; Ramos-Rodriguez et al., 2017).

In our opinion, LRGT helps in maintaining body weight, in line with previous studies showing that antidiabetic treatments may limit body weight loss in cachectic diabetic mice (Sugizaki et al., 2017; Infante-Garcia et al., 2018; Hierro-Bujalance et al., 2020). The LRGT treatment could also help in lowering glucose levels by increasing insulin secretion in diabetic mice (db/db and APP/PS1xdb/db) in the long term, as described previously (Qin et al., 2018), suggesting that LRGT also contributes in maintaining pancreatic activity in diabetic animals, in line with previous studies (Fan et al., 2018; Li et al., 2018).

We also observed that brain atrophy in diabetic animals was significantly reduced by long-term LRGT treatment. We specifically assessed the cortex, as this region is preferentially affected in APP/PS1xdb/db mice (Ramos-Rodriguez et al., 2015; Infante-Garcia et al., 2016). The LRGT treatment maintained cortical size and thickness in the APP/PS1xdb/db mice. On the other hand, we did not detect significant differences in the hippocampus, in line with previous studies showing that this region is affected later in the APP/PS1xdb/db mice (Ramos-Rodriguez et al., 2015; Infante-Garcia et al., 2016). Further assessment of NeuN/DAPI ratio also revealed that LRGT helps in maintaining neuron population in the long term. We also observed that LRGT reduced neuronal curvature, indicating an overall improvement in neuronal wellness, as reported previously, *postmortem* (Jackson et al., 2016; Infante-Garcia et al., 2017; Ramos-Rodriguez et al., 2017) and *in vivo* (Garcia-Alloza et al., 2007a; Meyer-Luehmann et al., 2008), reinforcing a neuroprotective role for LRGT in AD and T2D.

Glucagon-like peptide 1 receptors are mainly expressed in β-pancreatic cells and the gastrointestinal system, although they are also found in the brain (Hamilton and Hölscher, 2009; Farr et al., 2016). GLP-1, as a growth factor, increases cell growth and proliferation, and it also reduces neuronal injury and hippocampal apoptosis (During et al., 2003). Moreover, GLP-1 analogs induce cell proliferation and differentiation, and stimulate neurite growth (Salcedo et al., 2012). Similarly, the neuroprotective role of LRGT has been largely assessed in models has been shown to have a capacity to prevent synapse loss and deterioration of synaptic plasticity in AD (McClean et al., 2011; Batista et al., 2018), supporting further assessment of LRGT treatment in patients with mild Alzheimer’s dementia (Femminella and Edison, 2014). Other studies have also shown that LRGT enhances insulin sensitivity and improves insulin resistance (Kalra et al., 2010; Yamazaki et al., 2014; Tamura et al., 2015). Insulin resistance is not only a feature of diabetes pathology, but it is also an early alteration in AD, associated with basal elevations of insulin receptor substrate 1 (IRS1) phosphorylated in serine 616 (Talbot et al., 2012). Besides, serine phosphorylation of IRS1 is common in both AD and diabetes (Bomfim et al., 2012), and antidiabetic agents restore normal hippocampal formation responses to insulin in the IR–IRS-1–PI3K–Akt pathway (Bomfim et al., 2012). Similarly, LRGT significantly decreases IR aberrations in the APP/PS1 mice (Long-Smith et al., 2013). In our experiments, IR-A, IR-B, and IGF-1R mRNA expression levels were not significantly affected in APP/PS1xdb/db mice. While alterations at this level have been reported as a feasible link between AD and T2D (Holscher, 2021; Zheng and Wang, 2021), our studies did not include functional analysis, limiting the scope of our observations.

Liraglutide reduces tau hyperphosphorylation in both AD (Yang et al., 2013; Chen et al., 2017) and T2D models (Yang et al., 2013; Ma et al., 2015), indicating another feasible underlying mechanism for its neuroprotective role. While tau pathology is limited in the APP/PS1 mice, we observed a slight increase in tau phosphorylation both in the APP/PS1 and in db/db mice. Tau phosphorylation is significantly increased in the APP/PS1xdb/db animals, showing a synergistic effect when AD and T2D are set together (Ramos-Rodriguez et al., 2015; Infante-Garcia et al., 2016). Interestingly, early tau alterations, and not necessarily neurofibrillary tangles, might be critical for cognitive malfunctions (Hochgrafe et al., 2013), supporting the relevance of the AD-T2D crosstalk in tau pathology. We observed that the LRGT treatment reduces tau phosphorylation, as reported previously in AD models (Batista et al., 2018; Holubova et al., 2019; Jantrapirom et al., 2020), although to our knowledge the effects of LRGT on tau phosphorylation have not been assessed when AD-T2D coexist. On the other hand, the role of LRGT on amyloid pathology remains controversial, and previous studies have reported that LRGT has no effect on amyloid plaque burden in AD animals (Hansen et al., 2016). Our results are in line with observations reporting a reduction in amyloid pathology after LRGT treatment, such as significant reduction in the number of amyloid plaques (Holubova et al., 2019). LRGT also reverses amyloid plaque deposition (McClean et al., 2011; McClean and Hölscher, 2014), limits amyloid-related pathology (Batista et al., 2018; Jantrapirom et al., 2020), and even prevents amyloid deposition when administered prophylactically (McClean et al., 2015). While the burden of amyloid plaques in the APP/PS1xdb/db mice is lower than in the APP/PS1 animals (Ramos-Rodriguez et al., 2015; Infante-Garcia et al., 2016), an overall reduction of dense core plaque burden and plaque size is observed in the LRGT-treated mice. This is accompanied by a slight reduction in Aβ levels, which is in line with prior studies on 3×Tg AD mice (Duarte et al., 2020). We also detected a significant reduction in Aβ aggregates in the APP/PS1xdb/db animals, as reported previously, in the APP/PS1 mice (McClean et al., 2011). Given the relevance Aβ pathology in AD, further studies would be required to fully characterize Aβ structures and to provide a full picture of changes observed when AD and T2D coexist, as well as after LRGT treatment.

The anti-inflammatory properties of LRGT may also contribute to its neuroprotective effects (McClean et al., 2015; Hernández et al., 2016; Barreto-Vianna et al., 2017; Duarte et al., 2020). LRGT successfully reduced microglia activation in the proximity of amyloid plaques and in amyloid-free areas, showing a beneficial effect in both AD and T2D mice. The AD-T2D animals show an increase in microglia burden, mostly in areas free of amyloid plaques (Infante-Garcia et al., 2016), and LRGT significantly counterbalances this effect. The inflammatory response seems to be significantly disrupted when AD and T2D coexist, and metabolic disease cooperates to enhance the profiles of cytokines involved in neuronal injury, amyloid and tau pathologies, or blood-brain barrier damage (Sankar et al., 2020). In this sense, the db/db and, more severely, the APP/PS1xdb/db mice present small vessel disease that significantly improved after LRGT treatment. LRGT has positive effects on the peripheral vasculature (de Mesquita et al., 2017; Breton-Romero et al., 2018), and while studies on brain vasculature are more scarce, LRGT preserves blood-brain barrier integrity in a model of traumatic brain injury (Hakon et al., 2015). It also increases microvessel density and endothelial cell proliferation, reducing infarct brain volume after focal cortical ischemia (Chen et al., 2018), ultimately supporting a beneficial role of LRGT at the vascular level.

Spatial learning and memory improved after LRTG treatment in the MWM. Episodic memory was also enhanced in the new object discrimination test. Studies on patients show that episodic memory is affected early in AD (Ferguson and Alzheimer’s Disease Neuroimaging Initiative, 2021). Likewise, in patients with T2D-mild cognitive impairment, episodic memory correlated with glycated hemoglobin levels (Valenza et al., 2020). Our observations are in line with previous studies on AD models (McClean et al., 2011; McClean and Hölscher, 2014; Kamble et al., 2016; Chen et al., 2017) and diabetic mice (Yan et al., 2019). Clinical studies on the effects of LRGT on cognition are limited and controversial in some cases (for review Yaribeygi et al., 2021). Nevertheless, positive effects of LRTG and other GLP-1 analogs have been reported in prediabetic and diabetic patients (Vadini et al., 2020). Similarly, positive effects have been reported on patients with early AD and amnesic mild cognitive impairment (Watson et al., 2005), setting the basis for the ongoing assessment of LRGT in the ELAD trial (Femminella et al., 2019).

Our results support a positive role for LRGT at central level when AD and T2D coexist, as usually observed in the clinic (Janson et al., 2004). Brain atrophy, vascular damage, amyloid pathology, brain inflammation, and cognitive impairment are significantly ameliorated, supporting the beneficial role of LRGT in the brain and clinical studies on patients with AD.

## Data Availability Statement

The raw data supporting the conclusions of this article will be made available by the authors, without undue reservation upon reasonable request.

## Ethics Statement

The animal study was reviewed and approved by Junta de Andalucia (Guidelines for Care and Use of Experimental Animals, European Commission Directive 2010/63/UE and Spanish Royal Decree 53/2013) and the University of Cadiz Bioethics Committee.

## Author Contributions

MC-N and AM: experiment design, data acquisition, analysis, and interpretation. CH-B, PA-M, CI-G, MV-S, MH, BB-C, and IA-N: data acquisition and analysis. SL-L: design and critical revision of the manuscript for intellectual content. MG-A: study concept and design and drafting and critical revision of manuscript for intellectual content. All authors provided critical feedback and helped shape the research, analysis, and manuscript.

## Conflict of Interest

The authors declare that the research was conducted in the absence of any commercial or financial relationships that could be construed as a potential conflict of interest.

## Publisher’s Note

All claims expressed in this article are solely those of the authors and do not necessarily represent those of their affiliated organizations, or those of the publisher, the editors and the reviewers. Any product that may be evaluated in this article, or claim that may be made by its manufacturer, is not guaranteed or endorsed by the publisher.
