# Supplementary material for: Liraglutide Reduces Vascular Damage, Neuronal Loss, and Cognitive Impairment in a Mixed Murine Model of Alzheimer’s Disease and Type 2 Diabetes
Source: Front Aging Neurosci. 2021 Dec 16;13:741923. doi: 10.3389/fnagi.2021.741923 (PMC8716860; doi:10.3389/fnagi.2021.741923)
Supplement: Supplementary file 1 [file Data_Sheet_1.pdf]

Liraglutide reduces vascular damage, neuronal loss and cognitive impairment in a mixed murine model of Alzheimer´s disease and type 2 diabetes

Maria Jose Carranza-Naval[1,2,3], Angel del Marco[1,2], Carmen Hierro-Bujalance[1,2], Pilar Alves-Martinez[1,2], Carmen Infante-Garcia[1,2], Maria Vargas-Soria[1,2], Marta Herrera[1], Belen Barba-Cordoba[1], Isabel Atienza-Navarro[1,2], Simon Lubian-Lopez[2,4] and Monica Garcia-Alloza*[1,2]

[1]Division of Physiology. School of Medicine. Universidad de Cadiz. Cadiz, Spain.

[2]Instituto de Investigacion e Innovacion en Ciencias Biomedicas de la Provincia de Cadiz (INIBICA). Cadiz, Spain.

[3]Salus Infirmorum-Universidad de Cadiz. Cadiz, Spain.

[4]Division of Pediatrics. Section of Neonatology. Hospital Universitario Puerta del Mar. Cadiz, Spain.

*** Correspondence:**

Monica Garcia-Alloza

Division of Physiology

School of Medicine

Plaza Fragela sn, 4 piso 410

Tel. +34610022298

email. monica.garcia@uca.es

Supplementary information

Supplementary figure 1: Illustrative example of complete western blot for phospho-tau, total tau and β-actin in the cortex from all groups studied (Control, Control-LRGT, db/db, db/db-LRGT, APP/PS1, APP/PS1-LRGT, APP/PS1xdb/db and APP/PS1xdb/db-LRGT).

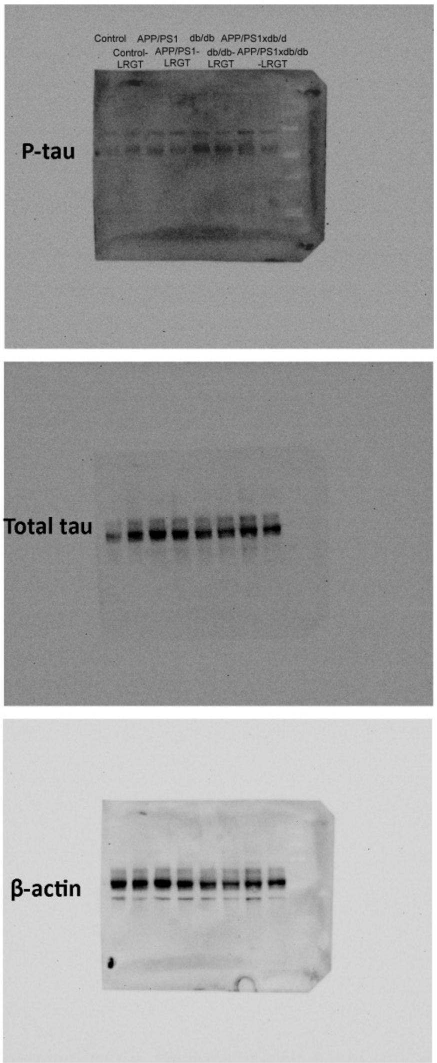

Figure legends with complete statistical study

Figure legends

Figure 1. Long-term LRTG treatment ameliorates metabolic alterations in T2D and AD-T2D mice. Non- fasted plasma glucose (a), insulin (b) and body weight (c) were measured every 4 weeks in controls, APP/PS1, db/db and APP/PS1xdb/db mice treated with vehicle or liraglutide (LGRT) at 500 μg/kg for 20 weeks (from week 6-week 26. **a)** LRGT significantly reduced postprandial glucose levels in diabetic mice: week 6 [$F_{(7,63)}$=4.79, ##p<0.01 Control, Control-LRGT, APP/PS1, APP/PS1-LRGT]; ╫╫p<0.01 vs. Control]; week 10 [$F_{(7,64)}$=15.74, †† p<0.01 Control, Control-LRGT, APP/PS1, APP/PS1-LRGT, db/db-LRGT and APP/PS1xdb/db-LRGT; ‡‡p<0.001 vs. Control, Control-LRGT, APP/PS1, APP/PS1-LRGT and db/db-LRGT]; ╤╤p<0.01 vs. Control-LRGT]; week 14 [$F_{(7,64)=}$14.065; ††p<0.01 vs. Control, Control-LRGT, APP/PS1, APP/PS1-LRGT, db/db-LRG and APP/PS1xdb/db-LRG; ╤╤p<0.01 vs. Control-LRGT]; week 18 [$F_{(7,64)}$=9.31, ††p<0.01 vs. Control, Control-LRGT, APP/PS1, APP/PS1-LRGT, db/db-LRGT, APP/PS1xdb/db-LRGT]; week 22 [$F_{(7,64)}$=8.14, ‡‡p=0.004 vs. Control, Control-LRGT, APP/PS1 and APP/PS1-LRGT]; week 26 [$F_{(7,60)}$=5.57, ##p<0.01 vs. Control, Control-LRGT, APP/PS1, APP/PS1-LRGT]. **b)** Insulin levels were maintained by long-term LRGT treatment. By 6 weeks of age, immediately before the commencement of LRGT treatment, insulin levels were significantly increased in APP/PS1xdb/db mice [F(7,64)=3.83, ##p<0.01 Control, Control-LRGT, APP/PS1, APP/PS1-LRGT]; week 10 [F(7,62)=5.42, ##p<0.01 Control, Control-LRGT, APP/PS1, APP/PS1-LRGT; week 14 [F(7,61)=10.53, ┴┴ p<0.01 vs. Control, Control-LRGT, APP/PS1, APP/PS1-LRGT, db/db and APP/PS1xdb/db; ╧╧p<0.01 vs. Control, Control-LRGT, APP/PS1, APP/PS1-LRGT, APP/PS1xdb/db]; week 18 [F(7,65)=10.75, ┴┴p<0.01 Control, Control-LRGT, APP/PS1, APP/PS1-LRGT, db/db and APP/PS1xdbdb; ##p<0.01 Control, Control-LRGT, APP/PS1, APP/PS1-LRGT]; week 22 [F(7,66)=10.60, ┴┴p<0.001 vs. Control, Control-LRGT, APP/PS1, APP/PS1-LRGT, db/db and APP/PS1xdb/db; ╧╧p<0.01 vs. Control, Control-LRGT, APP/PS1, APP/PS1-LRGT, APP/PS1xdb/db]; week 26 [F(7,66)=7.77, ┴┴ p<0.01 vs. Control, Control-LRGT, APP/PS1, APP/PS1-LRGT, APP/PS1xdb/db; ##p<0.01 vs. Control, Control-LRGT, APP/PS1 and APP/PS1-LRGT]. Differences detected by one-way ANOVA followed by Tukey b test. **c)** LRGT maintained body weight in APP/PS1-LRGT: week 6 [F(7,67)=18.57, ##p<0.01 vs. Control, Control-LRGT, APP/PS1 and APP/PS1-LRGT]; week 10 [F(7,67)=30.997, ##p<0.01 vs. Control, Control-LRGT, APP/PS1 and APP/PS1-LRGT]; week 14 [F(7,67)=33.39, ##p<0.01 vs. Control, Control-LRGT, APP/PS1 and APP/PS1-LRGT]; week 18 [F(7,67)=25.93, ##p<0.01 vs. Control, Control-LRGT, APP/PS1 and APP/PS1-LRGT], week 22 [F(7,66)=43.63, ┴┴p<0.01 vs. Control, Control-LRGT, APP/PS1, APP/PS1-LRGT and APP/PS1xdb/db, ##p<0.01 vs. Control, Control-LRGT, APP/PS1 and APP/PS1-LRGT]; week 26 [F(7,66)=37.44, ┴┴p<0.01 vs. Control, Control-LRGT, APP/PS1, APP/PS1-LRGT and APP/PS1xdb/db, ##p<0.01 vs. Control, Control-LRGT, APP/PS1 and APP/PS1-LRGT]. Differences detected by one-way ANOVA followed by Tukey b test. Data are representative of 5-12 animals and differences detected by one-way ANOVA followed by Tukey b or Tamhane tests.

Figure 2. LRGT treatment reduced cognitive impairment in APP/PS1xdb/db mice. Control, APP/PS1, db/db and APP/PS1xdb/db animals were analyzed in the new object discrimination test for "what", "when" and "where" paradigms (a) as well as in the

Morris water maze test (b, c). Behavioural assessment commenced on week 24, after 18 weeks of LRGT treatment (500 µg/kg/day), and was completed by week 26. **a)** LRGT improved episodic memory in the new object discrimination test. No differences were observed for “when” paradigm [$F_{(7,214)}=0.419$, p=0.890], however a significant improvement was observed for “what” [$F_{(7,194)}=2.76$, ††p=0.009 vs. Control, Control-LRGT and APP/PS1-LRGT] and “where” [$F_{(7,202)}=5.34$, **p<0.001 vs. rest of the groups] paradigms. **b)** LRGT also improved the performance along the acquisition phase in the MWM (day 1 [F(7,281)=4.65, ††p<0.01 vs. Control, Control-LRGT, APP/PS1, APP/PS1-LRGT, db/db and db/db-LRGT]; day 2 [F(7,273)=6.205, ##p<0.01 vs. Control, Control-LRGT, APP/PS1 and APP/PS1-LRGT, ŦŦp<0.01 vs. Control and Control-LRGT]; day 3 [F(7,282)=5.36, ##p<0.01 vs. Control, Control-LRGT, APP/PS1 and APP/PS1-LRGT, ŦŦp<0.01 vs. Control and Control-LRGT]; day 4 [F(7,277)=9.24, ‡‡p<0.01 vs. Control, Control-LRGT, APP/PS1, APP/PS1-LRGT, db/db-LRGT and APP/PS1xdb/db-LRGT, ##p<0.01 vs. Control, Control-LRGT, APP/PS1 and APP/PS1-LRGT, ŦŦp<0.01 vs. Control and Control-LRGT]). c) In the retention of the MWM we observed that LRGT treatment also improved the performance of APP/PS1xdb/db-LRGT mice [F(7,68)=3.63, ††p=0.002 vs. Control, Control-LRGT, and db/db-LRGT]. Data are representative of 5-12 animals.

Figure 3. Brain atrophy, neuronal density and curvature are reduced by LRGT treatment. Brain weight, cortex and hippocampal size (a, b), NeuN/DAPI ratio (c, d) and axonal curvature ratio (e, f) were analyzed in all 4 genotypes under study (Control, APP/PS1, db/db and APP/PS1xdb/db) and compared with animals by week 26, after 20 weeks on LRGT treatment (500 µg/kg/day). **a)** Long-term LRGT limited brain weight loss [$F_{(7,65)}= 6.27$, ┴┴p<0.01 vs. Control, Control-LRGT, APP/PS1, APP/PS1-LRGT, db/db and APP/PS1xdb/db]. Cortical size was significantly improved by LRGT treatment [$F_{(7,186)}=6.49$, ‡‡p<0.01 vs. Control, Control-LRGT, APP/PS1, APP/PS1-LRGT, db/db-LRGT and APP/PS1xdb/db-LRGT; ††p<0.01 vs. Control, Control-LRGT, APP/PS1 and APP/PS1-LRGT]. Differences did not reach statistical significance when hippocampus was analyzed [$F_{(7,95)}=0.16$, p=0.498]. Data are representative of 4-5 animals. **b)** Illustrative example of cresyl violet staining showing reduced cortical size in db/db and APP/PS1xdb/db mice. Scale bar=200 um. **c)** Neuronal density was reduced in the proximity of amyloid plaques in APP/PS1xdb/db mice and LRGT ameliorated this situation [$F_{(3,417)}=4.02$, ††p=0.008 vs. APP/PS1 and APP/PS1-LRGT]. A similar profile is observed in cortical and hippocampal areas with no amyloid plaques (cortex: [$F_{(3,4440)}=49.57$, ‡‡p<0.001 vs. Control, Control-LRGT, APP/PS1, APP/PS1-LRGT, db/db-LRGT, APP/PS1xdb/db-LRGT; ##p<0.01 vs. Control and Control-LRGT]; hippocampus: [$F_{(3,843)}=15.71$, ++p<0.01 vs. Control, Control-LRGT, APP/PS1, APP/PS1-LRGT, db/db and db/db-LRGT; ##p<0.01 vs. Control and Control-LRGT]). Data are representative of 5 animals (cortex 531-731 ROIs/group; hippocampus 60-200 ROIs/group). **d)** Illustrative example of NeuN (red) and DAPI (blue) staining in areas located in the proximity of amyloid plaques (TS staining, green) and in areas without amyloid plaques. Zoom-in images of representative regions are marked by white squares and presented next to the original image, including areas with and without amyloid plaques. Scale bar=50 µm, insets scale bar=25 µm. **e)** LRGT reduced curvature ratio in the proximity of amyloid plaques [$F_{(3,2084)}=4.46$, **p=0.004 vs. rest of the groups] and in areas free from amyloid plaques [$F_{(7,3498)}=13.13$, **p<0.01 vs. rest of the groups, ##p<0.01 vs. Control and Control-LRGT, ╫╫ p<0.01. vs. Control]. Data are representative of 5 animals per group (308-920 neurons/group). **f)** Illustrative examples of SMI-312 (red) and TS (green) staining

in the proximity and far from amyloid plaques (yellow lines mark representative neurites). Scale bar=10 μm.

Figure 4. LRGT affects amyloid and tau pathologies in APP/PS1xdb/db mice. Amyloid plaque burden and size are quantified by thioflavin S and 4G8 staining (a, b). Aβ40, Aβ42 and oligomeric Aβ levels are also determined in APP/PS1 and APP/PS1xdb/db mice after liraglutide treatment (500 μg/kg/day) for 22 weeks (c). Phospho-tau/total tau ratios are also measured in all genotypes under study (Control, APP/PS1, db/db and APP/PS1xdb/db) untreated or after LRGT treatement (d, e). **a)** LRGT treatment reduced amyloid plaques burden in the cortex from APP/PS1 mice (TS [$F_{(3,109)}$=10.025, **p<0.01 vs. rest of the groups]; 4G8 [$F_{(3,107)}$=5.60, ††p<0.001 vs. APP/PS1xdb/db and APP/PS1xdb/db-LRGT]. No differences were observed in the hippocampus (TS [$F_{(3,54)}$=1.21, p=0.312]; 4G8 [$F_{(3,55)}$=1.33, p=0.273]. LRGT treatment also reduced amyloid plaque size in the cortex from APP/PS1xdb/db mice (TS [$F_{(3,1246)}$=16.59, ‡‡p<0.01 vs. APP/PS1-LRGT and APP/PS1xdb/db-LRGT, ŦŦp<0.001 vs. APP/PS1-LRGT], 4G8[$F_{(3,2024)}$=17.34, **p<0.01 vs. rest of the groups, ##p<0.001 APP/PS1xdb/db-LRGT]). No differences were observed in the hippocampus (TS [$F_{(3,190)}$=2.44, p=0.065]; 4G8 [$F_{(3,314)}$=2.75, p=0.043, no further differences detected]). Data are representative of 5 animals. **b)** Illustrative image of TS (green) and 4G8 (red) staining of amyloid plaques in the cortex from all groups studied. Scale bar= 50 μm. **c)** As previously described soluble Aβ is slightly favored in APP/PS1xdb/db mice. LRGT treatment reduced soluble Aβ40 levels in the cortex although differences did not reach statistical significance [$F_{(3,25)}$=0.908, p=0.451]. No differences are observed in the cortex when soluble Aβ42 levels are analyzed [$F_{(3,26)}$=1.162, p=0.343]. However, oligomeric Aβ levels were significantly reduced in APP/PS1xdb/db mice on LRGT treatment [$F_{(3,24)}$=0.310, †p=0.046 vs. APP/PS1xdb/db]. No statistical differences were observed in the hippocampus for soluble Aβ40 [$F_{(3,26)}$=0.828, p=0.490] or Aβ42 levels [$F_{(3,24)}$=0.373, p=0.733]. Insoluble Aβ40 [$F_{(3,26)}$=3.62, ‡p=0.026 vs. APP/PS1] and Aβ42 [$F_{(3,24)}$=4.56, ‡p=0.011vs. APP/PS1] levels are reduced in APP/PS1xdb/db when compared with APP/PS1 animals, and LRGT treatment contributes to further reductions in the cortex. No differences were observed in the hippocampus for insoluble Aβ40 [$F_{(3,25)}$=0,743, p=0.537] or Aβ42 [$F_{(3,27)}$=0,207, p=0.891]. Data are representative of 6-9 animals. **d)** Tau phosphorylation was reduced in the cortex after LRGT treatment [$F_{(7,72)}$=2.97, ††p=0.009 vs. Control]. Differences did not reach statistical significance in the hippocampus [$F_{(7,34)}$=0.768, p=0.618]. Data are representative of 3-11 mice. **e)** Illustrative example of western blot for phospho-tau, total tau and β-actin in the cortex from all groups studied.

Figure 5. Spontaneous bleeding and inflammation are reduced after LRGT treatment while IR-A, IR-B and IGF-1R mRNA expression is not affected. Prussian blue staining is used to quantify hemorrhage burden and density in the cortex from untreated and treated (LRGT, 500 μg/kg/day) (a, b). Microglia burden was quantified by Iba1 immunostaining in the proximity (<50 μm) and far (>50 μm) from amyloid plaques in untreated and LRGT-treated animals (c, d). IR-A, IR-B and IGF-1R mRNA expression are also determined in the cortex from untreated and LRGT-treated mice (e). **a)** LRGT treatment reduces hemorrhage burden in the cortex [$F_{(7,156)}$=2.57, †p=0.012 vs. Control, Control-LRGT, APP/PS1, APP/PS1-LRGT, db/db-LRGT and APP/PS1xdb/db-LRGT]. A similar profile was observed when we analyzed cortical hemorrhage density [$F_{(7,159)}$=5.25, ††p<0.001 vs. Control, Control-LRGT, APP/PS1,

db/db-LRGT and APP/PS1xdb/db-LRGT]. No differences were detected in the hippocampus hemorrhage burden [$F_{(7,80)}$=0.403, p=0.898] or hemorrhage density [$F_{(7,82)}$=1.78, p=0.101]. Data are representative of 3-5 mice (489-1012 hemorrhages/group). **b)** Illustrative example of cortical hemorrhages stained with Prussian blue. Green arrows point at individual hemorrhages. Scale bar=100µm. **c)** LRGT treatment reduced cortical microglia burden in APP/PS1 and APP/PS1xdb/db mice, in the proximity of amyloid plaques [$F_{(3,527)}$=15.36, **p<0.01 vs. rest of the groups, ‡‡p<0.01 vs. APP/PS1]. LRGT also reduced microglia burden in cortical amyloid plaques free areas in diabetic mice [$F_{(7,4735)}$=81.81, **p<0.01 vs. rest of the groups, ††p<0.01 vs. Control, Control-LRGT, APP/PS1, APP/PS1-LRGT, db/db and db/db-LRGT, ₸₸p<0.01 vs. Control, Control-LRGT, APP/PS1, APP/PS1-LRGT and db/db, ‡‡p<0.01 vs. Control and Control-LRGT]. No statistical differences were observed in the hippocampus close to plaques [$F_{(3,30)}$=1.47, p=0.242] or far from amyloid plaques [$F_{(7,880)}$=1.14, p=0.335]. Data are representative of 5 mice (cortex 572-748 ROIs/group; hippocampus 108-230 ROIs/group). **d)** Illustrative example of cortical immunostaining for Iba1 (microglia, green) and 4G8 (amyloid plaques, red). Scale=100µm. Zoom-in images of representative regions are marked by white squares and presented next to the original image. Scale bar=50 µm, insets scale bar=10 µm. **e)** No differences were observed in the cortex when we analyzed IR-A [$F_{(7,51)}$=0.948, p=0.479], IR-B [$F_{(7,49)}$=0.504, p=0.827] or IGF-1R [$F_{(7,49)}$=0.735, p=0.644] mRNA expression. Data are representative of 6-9 mice.
